# Circadian regulation of metabolism across photosynthetic organisms

**DOI:** 10.1111/tpj.16405

**Published:** 2023-08-02

**Authors:** Luíza Lane de Barros Dantas, Bethany M. Eldridge, Jack Dorling, Richard Dekeya, Deirdre A. Lynch, Antony N. Dodd

**Affiliations:** ^1^ Department of Cell and Developmental Biology John Innes Centre, Norwich Research Park Norwich UK

**Keywords:** circadian regulation, metabolism, starch, rhizosphere, *Arabidopsis thaliana*, *Chlamydomonas reinhardtii*, *Ostreococcus tauri*, cyanobacteria, *Synechococcus elongatus* PCC7942, *Bacillus subtilis*

## Abstract

Circadian regulation produces a biological measure of time within cells. The daily cycle in the availability of light for photosynthesis causes dramatic changes in biochemical processes in photosynthetic organisms, with the circadian clock having crucial roles in adaptation to these fluctuating conditions. Correct alignment between the circadian clock and environmental day–night cycles maximizes plant productivity through its regulation of metabolism. Therefore, the processes that integrate circadian regulation with metabolism are key to understanding how the circadian clock contributes to plant productivity. This forms an important part of exploiting knowledge of circadian regulation to enhance sustainable crop production. Here, we examine the roles of circadian regulation in metabolic processes in source and sink organ structures of Arabidopsis. We also evaluate possible roles for circadian regulation in root exudation processes that deposit carbon into the soil, and the nature of the rhythmic interactions between plants and their associated microbial communities. Finally, we examine shared and differing aspects of the circadian regulation of metabolism between Arabidopsis and other model photosynthetic organisms, and between circadian control of metabolism in photosynthetic and non‐photosynthetic organisms. This synthesis identifies a variety of future research topics, including a focus on metabolic processes that underlie biotic interactions within ecosystems.

## Significance Statement

One way that circadian rhythms impact plant productivity is through their regulation of metabolism. Here, we explore this topic with a focus on the mechanisms of circadian control of metabolism, the circadian regulation of photoassimilate exudation into the rhizosphere, and shared and differing aspects of the circadian regulation of metabolism across photosynthetic organisms.

## INTRODUCTION

The metabolism of plants underlies the productivity of our ecosystems and crops. Plants require sunlight for photosynthesis, meaning that metabolic regulation is intimately associated with 24 h fluctuations in environmental conditions. This is thought to have been selected for extensive circadian regulation of metabolism, explaining some of the links between the circadian clock and plant growth. Therefore, the circadian regulation of metabolism is crucial for the performance of both crops and natural plant populations. There is considerable spatial and phylogenetic heterogeneity of metabolism in photosynthetic organisms, such as the metabolic dependence of plant roots upon the leaves, and key differences in storage carbohydrates across the green lineage.

Circadian clocks have been described in plants, animals, fungi, photosynthetic and non‐photosynthetic bacteria. These clocks have many shared regulatory principles and characteristics, yet differ in their molecular composition between taxonomic groups. Here, we examine the nature of the circadian regulation of metabolism by comparing contrasting plant structures of the model plant *Arabidopsis thaliana* (Arabidopsis) and extend our comparisons to photosynthetic and non‐photosynthetic model organisms (Box 1). We also argue that circadian regulation of plant metabolism has a key role in the interactions that occur between plants and their biotic environments (Box 1).

Box 1 **Main points of review**

- There is extensive circadian regulation of metabolism in plants.
- Circadian regulation of metabolic processes might influence interactions between plants and the rhizosphere.
- Key principles of the circadian regulation of metabolism are shared across photosynthetic organisms.
- There are conceptual similarities and differences between the circadian regulation of metabolism in plants and non‐photosynthetic organisms.

## GENERATION OF CIRCADIAN RHYTHMS IN PLANTS

**Figure 1:**
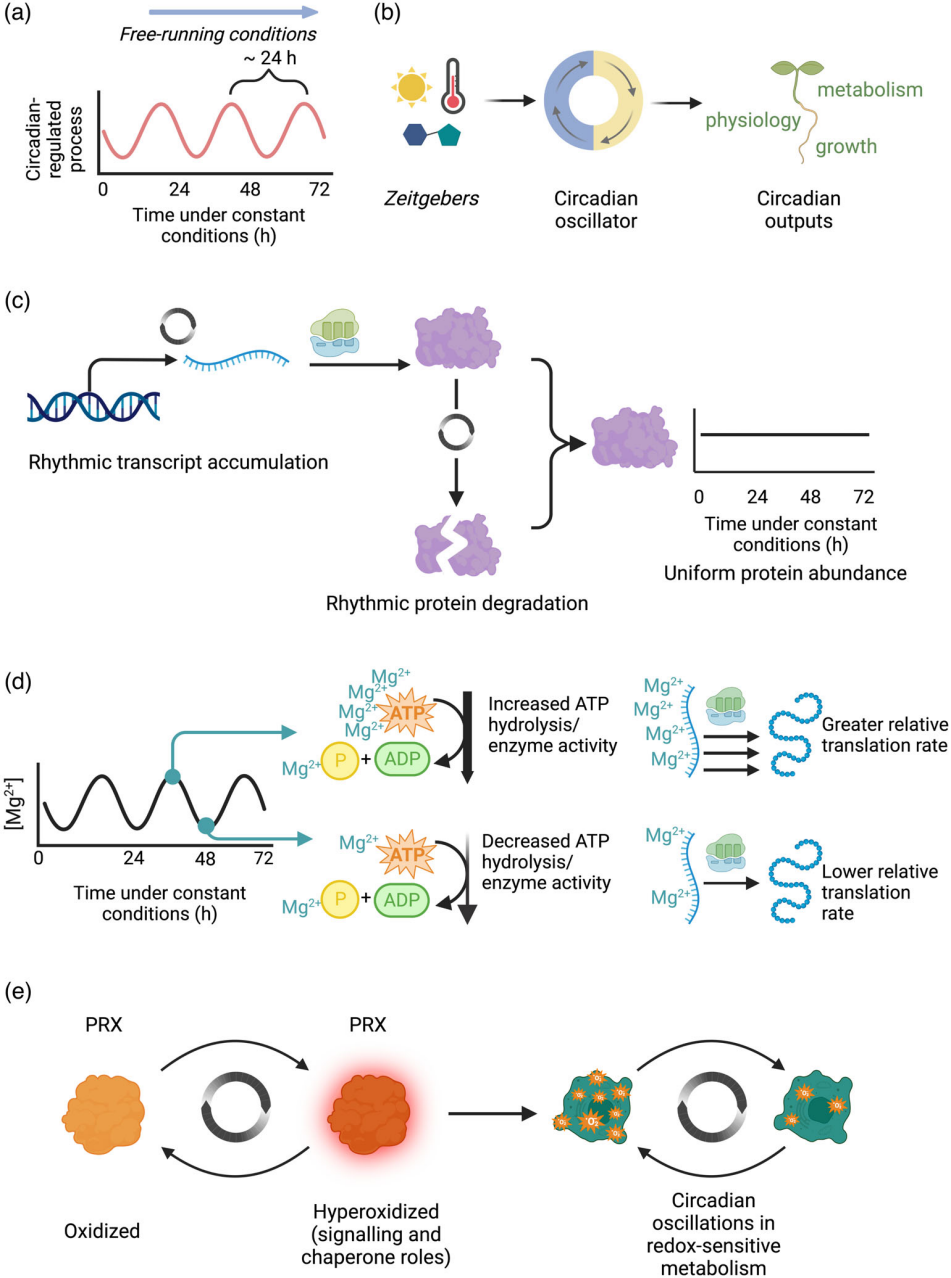
Processes of circadian regulation. (a) Circadian rhythms are endogenous biological cycles that have a period of approximately 24 h under constant conditions. Under appropriate free‐running conditions, the rhythms persist in the absence of external cues such light or temperature. (b) The circadian oscillator of Arabidopsis is entrained to several environmental cues (known as zeitgebers) that align its phase with environmental fluctuations. The circadian oscillator produces an estimate of time, which is communicated to clock‐controlled processes through transcriptional and post‐transcriptional mechanisms. (c) A regulatory architecture underlying extensive circadian regulation of transcript abundance in the absence of equivalent extensive oscillations in protein abundance. Here, circadian oscillations of transcript accumulation permit oscillations of protein replacement, leading to proteostasis. (d) Circadian oscillations in the concentration of Mg^2+^ (Feeney et al., 2016) might lead to wide‐scale oscillations in ATP hydrolysis and translation rates, driving circadian rhythms of metabolism. (e) Circadian oscillations in the oxidation state of peroxiredoxin (PRX) (Edgar et al., 2012) might drive or be related to circadian rhythms of redox‐sensitive metabolism. In the hyperoxidized state, PRX has signaling and chaperone functions, and these oscillations might occur independently or semi‐independently from the transcription‐translation feedback loops (Edgar et al., 2012). In (c–e), the gray circular icon indicates points of circadian control.

Circadian rhythms are self‐sustained biological cycles that have a period of around 24 h under constant conditions (free running conditions; Figure 1a). In plants, circadian rhythms are generated by the circadian oscillator, which is a network of transcriptional regulators that are organized into a set of feedback loops (the transcription–translation feedback loop [TTFL] based oscillator). There is also a redox‐based oscillator, which might function independently from the TTFL (Edgar et al., 2012; O'Neill et al., 2011). Information concerning light, temperature, and metabolic conditions is communicated to the circadian oscillator, to align its phase with the environmental conditions, through the process of entrainment (Figure 1b). Environmental cues that lead to entrainment are known as zeitgebers (Figure 1b). The estimate of time that is produced by the circadian oscillator is communicated to clock‐controlled processes through “output pathways” (Figure 1b), through mechanisms including transcriptional regulation.

We briefly describe the architecture of the Arabidopsis circadian oscillator, referring readers elsewhere for further detail (Hsu & Harmer, 2014; Millar, 2016). During the morning, transcripts encoding MYB‐like transcription factors *CIRCADIAN CLOCK‐ASSOCIATED1* (*CCA1*) and *LATE ELONGATED HYPOCOTYL* (*LHY*) and their concomitant proteins accumulate to a high level (Alabadí et al., 2001). CCA1 and LHY repress the accumulation of transcripts encoding *TIMING OF CAB EXPRESSION1* (*TOC1*) (Alabadí et al., 2001). In turn, the accumulation of transcripts encoding *CCA1* and *LHY* is repressed by TOC1 in a transcriptional feedback loop (Gendron et al., 2012; Huang et al., 2012). CCA1 and LHY also repress the accumulation of transcripts of *GIGANTEA* (*GI*), *LUX ARRYTHMO* (*LUX*), *BROTHER OF LUX ARRHYTHMO* (*BOA* or *NOX*), *EARLY FLOWERING3* (*ELF3*), and *ELF4* through promoter binding (Dai et al., 2011; Hsu & Harmer, 2014). Evening gene repression by CCA1 and LHY depends upon DEETIOLATED1 (DET1), a key repressor in photomorphogenesis (Lau et al., 2011). *PSEUDO‐RESPONSE REGULATOR9* (*PRR9*), *PRR7* and *PRR5* are homologs of *PRR1* (which is *TOC1*), and their transcripts accumulate during the day (Farré & Liu, 2013). *PRR9* transcripts reach peak levels just before dawn, followed by *PRR7*, *PRR5* and *PRR3*, and *TOC1* (Farré & Liu, 2013). PRR9, PRR7, and PRR5 have partially redundant roles in the repression of *CCA1* and *LHY* (Nakamichi et al., 2010). The PRRs also repress the expression of *REVEILLE8* (*RVE8*) (Rawat et al., 2011).

## SPATIOTEMPORAL REGULATION OF PRIMARY METABOLISM

The circadian regulation of metabolism is particularly well characterized in Arabidopsis (Bläsing et al., 2005; Haydon et al., 2013; Usadel et al., 2008), although other good models for source‐sink dynamics include *Brachypodium distachyon* (Kellogg, 2015), wheat (Lawlor & Paul, 2014), and the C_4_ species *Setaria viridis* and maize (Martin et al., 2016; Rosado‐Souza et al., 2023), Here, we use studies of Arabidopsis to examine the circadian regulation of primary metabolism within plant organs functioning as a carbon source (e.g., leaves) and photoassimilate sinks (e.g., roots). We reason that roles for circadian regulation within these structures differ. For example, the shoot circadian oscillator is thought to dominate over that of roots (James et al., 2008; Nimmo, 2018; Takahashi et al., 2015). Small molecules such as sucrose can be transported from leaves to roots in Arabidopsis, conveying temporal information and acting as agents of long‐distance circadian regulation (Chen, Qu, et al., 2012; Gottwald et al., 2000; Tong et al., 2022).

The circadian regulation of starch anabolism and catabolism is one of the better‐understood aspects of circadian oscillator‐mediated metabolic regulation in Arabidopsis. Whilst both leaves and roots synthesize and degrade starch (Lu et al., 2006; Malinova et al., 2011), roots maintain sufficiently low transitory starch levels that this is thought unlikely to sustain growth or provide carbon skeletons for metabolism (Malinova et al., 2011; Pignocchi et al., 2021). Leaf transitory starch abundance follows a carefully‐regulated daily rhythm under cycles of light and dark. A high transitory starch level is reached at the end of the photoperiod, and this is consumed in a regulated manner during the night to reach a low level at dawn without exhausting reserves completely (Graf et al., 2010). This 24 h dynamic is regulated by the circadian clock under cycles of light and dark, and explains some of the biomass accumulation and growth phenotypes of circadian clock mutants (Chew et al., 2014; Dodd et al., 2005; Graf et al., 2010). Further contributors might include optimal rates of photosynthesis when the circadian period and environmental cycles are aligned (Dodd et al., 2005), and the widespread contributions of circadian regulation to programs of gene expression (Harmer et al., 2000). When wild‐type Arabidopsis plants experience unexpected early or late dusk, the rate of starch degradation is adjusted accordingly (Graf et al., 2010; Lu et al., 2005).

There is a bidirectional relationship between the circadian oscillator and primary metabolism. Primary metabolites entrain the circadian oscillator by adjusting its phase (Frank et al., 2018; Haydon et al., 2013). These metabolites work partly as indirect zeitgebers for light because photosynthesis is a light‐driven process. However, they also provide information to the circadian oscillator about changes in carbohydrate availability during the dark period (Webb et al., 2019). It is thought that the transcription factor bZIP63 binds the promoter of the circadian oscillator gene *PRR7*, regulating its expression and circadian phase in Arabidopsis leaves in response to the regulation of bZIP63 by sucrose (Frank et al., 2018). This occurs through another metabolite, the signaling sugar trehalose‐6‐phosphate (Tre6P), which is thought to signal sucrose status to bZIP63 through a process involving SNF1‐RELATED PROTEIN KINASE1 (Frank et al., 2018). Tre6P is a signaling molecule to plant cells that participates in signaling of sugar status (Figueroa & Lunn, 2016).

There are circadian rhythms in the abundance of transcripts encoding key enzymes for starch anabolism and catabolism (Covington et al., 2008; Harmer et al., 2000), suggesting that circadian regulation might act upon starch metabolism at the transcriptional level. Transcripts encoding ADP GLUCOSE PYROPHOSPHORYLASE1 (APS1), chloroplast‐localized phosphoglucose mutase (PGM1) and STARCH BRANCHING ENZYME2.2 (SBE22) accumulate with a circadian rhythm (Covington et al., 2008). Of these, ADP‐glucose pyrophosphorylase is a key enzyme for starch biosynthesis that catalyzes the first and rate‐limiting step in this pathway (Okumura et al., 2016). PGM1 connects starch anabolism to the Calvin‐Benson cycle by regulating carbon flow (Stitt & Zeeman, 2012), so there is circadian regulation of a transcript at a key point of metabolic convergence. Within the starch degradation pathway, there is circadian accumulation of several transcripts: α‐glucan water dikinase (*GWD1* or *SEX1*), phosphoglucan water dikinase (*PWD*), debranching enzyme 3 (*ISA3*), debranching enzyme 4 (*ATPU1*), alpha‐glucan phosphorylase 2 (*PHS2*), disproportionating enzymes 1 (*DPE1*) and *DPE2*, and starch excess 4 (*SEX4*) (Harmer et al., 2000). Of particular note are the transcripts encoding GWD and PWD, whose products catalyze early steps of starch degradation (Usadel et al., 2008). *SEX4* transcripts are expressed at very low levels in the circadian oscillator triple mutant *rve468*, leading to an increased starch content phenotype (Scandola et al., 2022). This starch excess phenotype also occurs in the *prr7 prr9* double mutant (Chew et al., 2022; Flis et al., 2019). The arrhythmic *prr5 prr7 prr9* triple mutant has a greater accumulation of transcripts associated with starch biosynthesis and degradation (Nakamichi et al., 2012), although it is not clear whether starch content is altered in this mutant. It would be informative to establish the extent to which transcripts associated with starch metabolism are rhythmic in roots, to better understand the role of the clock in transitory starch management within roots.

As the carbon for primary root growth derives from leaves (Koch, 2004), sucrose transport is necessary for its delivery to roots. Transcripts encoding sucrose transporters from the AtSUC and AtSWEET families are rhythmic, such as *SWEET11* expressed in leaves, and *SWEET14* expressed in roots (Covington et al., 2008; Durand et al., 2018). Transcripts encoding the *SUC1* transporter that unloads sucrose in roots are also rhythmic (Covington et al., 2008; Durand et al., 2018). If the abundance of these transcripts influences protein abundance or activity, the loading/unloading of sucrose might also be circadian‐regulated. Another important class of proteins associated with sucrose metabolism are the neutral and acid invertases, which are part of the sucrose catabolic pathway. Transcripts encoding CYTOSOLIC INVERTASE1 (CINV1) are rhythmic (Covington et al., 2008; Harmer et al., 2000) and the loss‐of‐function *cinv1* and *cinv2* mutants have reduced root growth and cell size, accompanied by altered primary metabolism and signs of carbon starvation (Pignocchi et al., 2021). Overall, photoassimilated carbon in leaves is transported to roots and there is evidence of circadian regulation of transcripts associated with multiple steps of this process, from synthesis to transport unloading. This aligns with the view that there is communication between circadian oscillators in leaves and roots, with hierarchical control from leaves to roots (James et al., 2008; Nimmo, 2018; Takahashi et al., 2015; Uemoto et al., 2023). There are various hypotheses concerning the molecular signal(s) that travel from leaves to roots to fulfill this role. Several non‐mutually exclusive possibilities include circadian oscillator components (e.g. mobile ELF4; Chen et al., 2020), light piping (Nimmo, 2018), metabolic signals such as mobile carbohydrates (e.g. sucrose or Tre6P), or mobile ions (James et al., 2008; Uemoto et al., 2023). It will be informative in the future to determine whether these putative signals form part of a single regulatory process, or whether they have distinct roles related to different environmental conditions or developmental stages.

## TRANSCRIPTIONAL AND POST‐TRANSCRIPTIONAL CIRCADIAN CONTROL OF METABOLISM

Although there is extensive circadian regulation of transcript abundance in Arabidopsis (Covington et al., 2008; Harmer et al., 2000), protein abundance is often not altered accordingly (Choudhary et al., 2015; Uhrig et al., 2021). Therefore, the interpretation of transcript abundance data in the context of metabolic regulation requires caution. One function of the circadian regulation of transcript accumulation could be to replenish proteins that are turned over with a 24 h cycle, to maintain proteostasis (Figure 1c) (Seinkmane et al., 2022). There are also circadian rhythms in the phosphorylation state of a variety of metabolic proteins (Choudhary et al., 2015; Krahmer et al., 2022). For example, in CAM models the circadian oscillator controls transcript levels and activity of the regulatory protein kinase phosphoenolpyruvate carboxylase kinase (PPCK) that regulates the activity of CO_2_‐fixing PEP carboxylase (Hartwell et al., 1996; Taybi et al., 2000).

Post‐translational mechanisms might broadcast circadian timing information to multiple cellular processes. For example, there are circadian oscillations in the concentration of Mg^2+^ (Feeney et al., 2016). These might act as a general circadian regulatory mechanism of metabolic processes involving ATP hydrolysis, and global translation rates (Feeney et al., 2016) (Figure 1d). This might explain why Mg^2+^ can also alter the circadian period length in both algae and Arabidopsis (de Melo et al., 2021; Feeney et al., 2016). Furthermore, rhythms in the concentration of Mg^2+^ in rice under light/dark cycles have been implicated in the regulation of the rate of photosynthesis, with the rice orthologs of certain *PRR* genes implicated in the regulation of this process (Chen et al., 2022; Li et al., 2020). There is also circadian regulation of several markers of the redox state that could present broad‐spectrum circadian regulators of metabolic processes (Figure 1e) (Edgar et al., 2012; Lai et al., 2012; Zhou et al., 2015). It would be informative also to know whether the process of methylation can act as a general clock‐controlled regulator of metabolism in plants, given that it affects circadian clock function in photosynthetic organisms (Fustin et al., 2020). This might occur through its role in RNA processing, as demonstrated in mammals (Fustin et al., 2013). Since several of these processes can be circadian‐regulated in the absence of a functional transcription‐translation circadian oscillator (Figure 1e) (Edgar et al., 2012; Feeney et al., 2016; Krahmer et al., 2022), they might represent ancient forms of circadian metabolic control that are conserved across several domains life.

## CIRCADIAN REGULATION OF PHOTOSYNTHESIS IN EUKARYOTES

In flowering plants, aspects of photosynthetic metabolism are circadian regulated. Circadian rhythms in net CO_2_ uptake occur in species including *Vicia faba* and Arabidopsis (Dodd et al., 2004, 2005; Hennessey & Field, 1991). These rhythms in Arabidopsis appear to be regulated by the canonical circadian oscillator because circadian oscillator mutants that alter the period, and overexpressors causing arrhythmia, lead to corresponding changes in net CO_2_ assimilation (Dodd et al., 2004, 2005). Furthermore, chlorophyll fluorescence methodologies such as delayed fluorescence (Gould et al., 2009), prompt fluorescence (Dakhiya et al., 2017), and pulse‐amplitude modulated fluorescence (Dakhiya et al., 2017) suggest that the circadian oscillator controls the light reactions of photosynthesis.

The circadian regulation of photosynthesis appears to be conserved across the green lineage because this occurs also in non‐flowering plants and algae. For example, circadian regulation of delayed chlorophyll fluorescence occurs in the bryophyte *Physcomitrium patens* (*Physcomitrella*) (Gyllenstrand et al., 2014), and both delayed fluorescence and PAM fluorescence are rhythmic in *Marchantia polymorpha* (Cuitun‐Coronado et al., 2022). Furthermore, circadian rhythms of photosynthesis have been reported in the algae *Gonyaulax polyedra* (measuring carbon assimilation, Hastings et al., 1961), *Acetabularia major* (measuring O_2_ evolution; Sweeney & Haxo, 1961), and *Aegagropila linnaei* (measuring delayed fluorescence and PAM fluorescence; Cano‐Ramirez et al., 2018). However, in certain gymnosperms such as *Picea abies*, circadian rhythms of delayed chlorophyll fluorescence have not been detected (Gyllenstrand et al., 2014). The mechanisms underlying the circadian regulation of photosynthesis have remained somewhat elusive, and may involve extensive circadian control of photosynthetic gene expression (Harmer et al., 2000; Millar et al., 1992; Pilgrim & McClung, 1993), circadian signaling to chloroplasts (Noordally et al., 2013), circadian regulation of RuBP supply and Rubisco activation state (Fredeen et al., 1991; Liu, Taub, et al., 1996), redox regulation, and the concentration of ions such as Mg^2+^ (Chen et al., 2022; Feeney et al., 2016). In addition to Mg^2+^, Ca^2+^ signals within chloroplasts contribute to the regulation of photosynthesis (Frank et al., 2019; Terashima et al., 2012) and there are circadian oscillations in the concentration of free Ca^2+^ within chloroplasts (Johnson et al., 1995), suggesting a further mechanism that might couple the circadian oscillator with photosynthetic metabolism.

Two fascinating photosynthetic specializations for which temporal control is important are Crassulacean acid metabolism (CAM) and C_4_ photosynthesis. Whilst circadian regulation is relevant to both CAM and C_4_ photosynthesis, we have chosen to focus on CAM because there have been interesting recent developments in this field. In CAM plants, atmospheric CO_2_ is fixed initially by phosphoenolpyruvate carboxylase (PEPC) during the night and stored temporarily as organic acids such as malate. During the day, the organic acids are decarboxylated behind closed stomata and the carbon undergoes secondary fixation by Rubisco, powered by photosynthetic light harvesting. This temporal separation of two carbon fixation steps permits nocturnal stomatal opening when the relative humidity is high and temperatures are lower, increasing the water use efficiency relative to C_3_ species. Accordingly, CAM is often present in species occupying environments with low water availability such as deserts, salt marshes, and epiphytic niches. Circadian regulation has a key role in the temporal separation of activity of the two carboxylases. PEPC is inhibited by malate, but its phosphorylation by PPCK renders it insensitive to this inhibition. During the night, circadian regulation increases *PPCK* transcript levels, which increases PPCK activity and phosphorylates PEPC, leading to its activation (Carter et al., 1991; Nimmo et al., 1987). During the photoperiod, PEPC dephosphorylation leads to its inhibition by malate, and the stored malate is decarboxylated for refixation by Rubisco. In addition to this 24 h cycle in the regulation of PEPC, multiple CAM cycle components (e.g. organic acid transport into and out of the vacuole, diurnal organic acid decarboxylation, control of stomatal aperture) require temporal coordination for the CAM metabolic syndrome to function effectively. Therefore, an important focus has been to understand the relative contributions to CAM of the circadian regulation of its many components. PEPCK is circadian‐regulated at the level of transcript abundance (Hartwell et al., 1996, 1999; Taybi et al., 2000), and 24 h cycles in its expression are necessary for the daily cycle of PEPC phosphorylation, maximum rates of nocturnal CO_2_ fixation, and circadian regulation of CO_2_ fixation (Boxall et al., 2017). Intriguingly, misexpression of each of PEPCK and PEPC alters the accumulation of some circadian clock transcripts (Boxall et al., 2017, 2020), but it is unclear whether this produces an overall alteration in the circadian rhythm. In addition to nocturnal CO_2_ fixation, silencing each of two enzymes associated with the diurnal decarboxylation of accumulated malate (mitochondrial NAD‐malic enzyme (NAD‐ME) and cytosolic/plastidic pyruvate orthophosphate dikinase) reduces nocturnal malate accumulation and abolishes nocturnal CO_2_ fixation (Dever et al., 2015). Under free‐running conditions, this damps the circadian oscillation of net CO_2_ uptake and abolishes the circadian cycle of PPCK phosphorylation that is necessary for nocturnal CO_2_ fixation (Dever et al., 2015). Similar to the misexpression of PEPC and PPCK, silencing NAD‐ME alters the circadian rhythm of expression of certain clock components, in this, case *TOC1* (Boxall et al., 2017, 2020; Dever et al., 2015). Taken together, this suggests there is metabolic feedback from the CAM cycle to the circadian clock, which is reminiscent of the metabolic regulation of the circadian clock in Arabidopsis (Haydon et al., 2013). This might provide one mechanism that integrates circadian and metabolic information to confer plasticity to the CAM cycle, in response to variations in environmental conditions (Borland et al., 1999; Dodd et al., 2002). There is extensive rephasing of a variety of other processes in some CAM species compared with Arabidopsis, including redox metabolism (Abraham et al., 2016), suggesting that system‐wide changes in the relationship between the clock and metabolism formed an important part of the evolution of the CAM syndrome.

## CIRCADIAN REGULATION OF ROOT EXUDATION

Between 10% and 40% of photoassimilated carbon and 15% of nitrogen is allocated below‐ground (Haichar et al., 2016; Kuzyakov & Domanski, 2000; Whipps, 1990), where it is exuded into the environment as a dynamic array of compounds known as root exudates. These comprise many low and high‐molecular weight metabolites that are passively and actively released by sloughing, diffusion, exocytosis, and transmembrane proteins (see the excellent reviews of Badri & Vivanco, 2009; Galloway et al., 2018; Oburger & Jones, 2018). Root exudation shapes the biotic and abiotic properties of the root‐environment interface (the rhizosphere), and contributes to plant fitness by regulating a variety of physiochemical and biological interactions such as soil aggregation, water and nutrient uptake, and signaling to the complex community of rhizosphere‐dwelling organisms (the rhizomicrobiome) (Badri & Vivanco, 2009; Baetz & Martinoia, 2014; Mommer et al., 2016; Oburger & Jones, 2018; Sasse et al., 2018). In terms of mitigation of global climate change, root exudation is an important pathway for carbon capture and below‐ground storage (Panchal et al., 2022).

**Figure 2:**
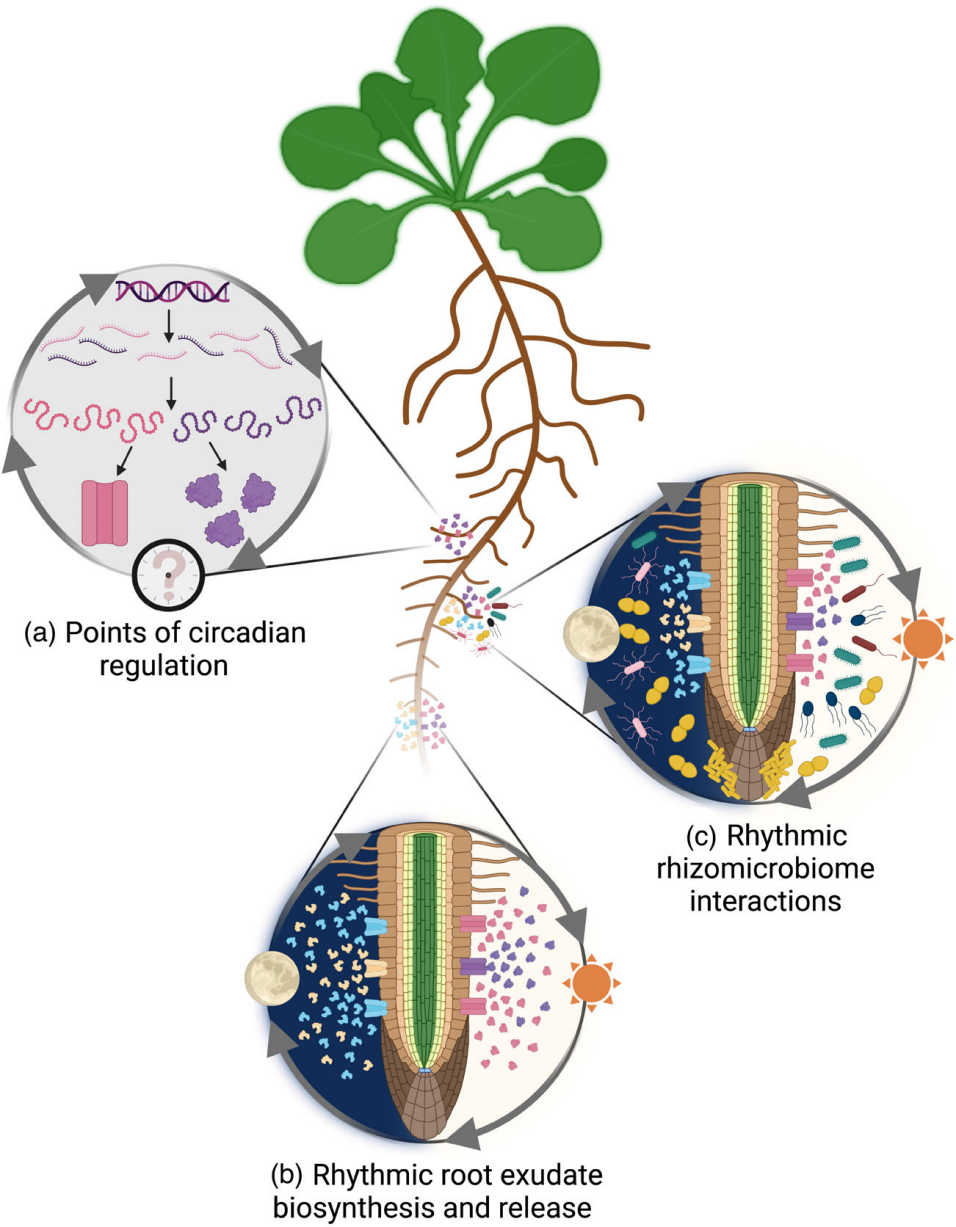
Potential involvement of the circadian oscillator in rhythmic root exudation and rhizosphere interactions. (a) Circadian‐regulated rhythmicity in root metabolite biosynthesis and transport might directly or indirectly affect root exudation processes at the transcriptional, translational, and post‐translational scales. (b) The circadian oscillator is likely to contribute to diel fluctuations in root metabolite release into the rhizosphere. (c) Rhythmic changes in root exudation might affect microbial community composition, which could drive rhythmic metabolic feedback, shaping rhizosphere structure and function.

The mechanistic dynamics of the spatiotemporal release of root metabolites governs biotic and abiotic processes in the rhizosphere (Darwent et al., 2003; Kuzyakov & Razavi, 2019; McDougall & Rovira, 1970; Roque‐Malo et al., 2020; Spohn & Kuzyakov, 2014; Van Egeraat, 1975; Weidenhamer et al., 2014). Given that the circadian oscillator controls multiple areas of plant metabolism (Bläsing et al., 2005; Fukushima et al., 2009; Harmer et al., 2000), it is reasonable to hypothesize that root exudation will be circadian‐regulated. However, the mechanistic role of circadian regulation in root exudate biosynthesis and transport remains poorly understood. A recent study provided a potential link between the circadian clock and exudate release in Arabidopsis by comparing the root metabolite profiles of the wild type and circadian clock mutants *toc1‐*101 and *cca1‐*1 (Lu et al., 2021). By cumulatively sampling root exudates from soil at five timepoints over 24 h, *toc1‐*101 and *cca1‐*1 were found to produce fewer rhythmic root exudate compounds (45 and 39, respectively) compared with the wild type (50; Lu et al., 2021). The five top rhythmic exudates in the wild type (identified as lipid and lipid‐like molecules) were reported to be arrhythmic in these clock mutants (Figure 2a; Lu et al., 2021).

Indirect evidence linking circadian rhythms and root exudation derives from studies of plants grown under light/dark (diel) cycles (Matsuda et al., 2020; McLaughlin et al., 2023; Oburger et al., 2014; Reichman & Parker, 2007; Selby‐Pham et al., 2017; Tixier et al., 2023). Phytosiderophores, which are important for iron mobilization from soil, are released from the roots of *Oryza sativa* (rice; Selby‐Pham et al., 2017), *Hordeum vulgare* (barley; Mori et al., 1987; Takagi et al., 1984; Walter et al., 1995), *Triticum aestivum* (wheat; Oburger et al., 2014; Reichman & Parker, 2007), *Zea mays* (maize; Ueno et al., 2009) and *Festuca rubra* (red fescue; Ma et al., 2003) during the day. Diel cycles of exudation of metabolites including simple sugars and amino acids has been reported in *Pisum sativum* (pea; Tixier et al., 2023), and secondary compounds including isoflavones, flavonoids and glucosinolates from *Glycine max* (soybean; Matsuda et al., 2020) and Arabidopsis (Badri et al., 2010). A recent report described day‐to‐night fluctuations in the abundance of 7–32% of exudates released from Arabidopsis, *Brachypodium distachyon*, and *Medicago truncatula* (McLaughlin et al., 2023). Most of these compounds had greater abundance at the end of the day than at the end of the night (McLaughlin et al., 2023). Whether the diel cycles of these exudates are regulated by the circadian clock is yet to be determined.

Comparison of the microbial communities inhabiting the rhizosphere of wild‐type, clock mutant, and clock gene‐overexpressing Arabidopsis has revealed differences between the rhizomicrobiome community structure of these genotypes (Hubbard et al., 2018, 2021; Newman et al., 2022; Staley et al., 2017). Collectively, these studies imply a role for the circadian oscillator in root metabolite release, because exudation shapes rhizomicrobiome community structure (e.g. Haichar et al., 2008; Hugoni et al., 2018; Kawasaki et al., 2021; Shi et al., 2011; Ulbrich et al., 2022; Zhao et al., 2021). Future research to disentangle feedback loops between rhythmic exudate production and rhizomicrobiome composition will enable the assessment of the ecological context of exudate rhythmicity and root–environment communication.

Whilst these studies establish the importance of temporal regulation in root exudation, we are only beginning to understand how the circadian oscillator shapes this process. To address this knowledge gap, we propose four broad topics for future research:

*Disentangle the direct and indirect role of circadian regulation in root exudation*: The direct influence of the circadian oscillator on biosynthesis and transport pathway(s) needs to be understood mechanistically for key exudate compounds (Figure 2a). For example, the circadian oscillator could directly regulate the production of certain metabolite(s) or transport protein(s). Alternatively, the circadian oscillator could indirectly regulate exudate release by affecting the availability of cofactors required for exudate biosynthesis (e.g., Mg^2+^ and ATP; Figures 1d, 2c) or through regulation of transport processes. For example, circadian regulation has been reported within the root endodermal barriers that are important for apoplastic transit (Durr et al., 2021), and is involved in protein phosphorylation events that could regulate the activation state or substrate preference of membrane‐localized exudate transporters (Choudhary et al., 2015; Krahmer et al., 2022; Kusakina & Dodd, 2012; Prado et al., 2019).
*Evaluate the levels at which the circadian oscillator regulates rhythmicity in root exudate biosynthesis and release*: Investigations of circadian rhythmicity of root metabolite biosynthesis and transport have focused on transcript abundance (Figure 2a) (Badri et al., 2010; Lu et al., 2021; Matsuda et al., 2020). Future studies will benefit from evaluating whether the circadian oscillator governs root metabolite production and release at the levels of protein activity, metabolite accumulation, and metabolite transport.
*Assess root exudate rhythmicity across various plant developmental stages and environments*: Root exudate composition changes with plant development stage (Chaparro et al., 2013) and growing substrate (McLaughlin et al., 2023), so comparative experiments using wild‐type and circadian clock mutants at different developmental stages, using various substrates, will deepen understanding of the circadian regulation of root exudation over the plant life cycle and its influence by the environment. Such investigations will benefit from non‐targeted root exudate profiling approaches to obtain a holistic assessment of circadian involvement in the release of different metabolite classes, because identification and quantification of various compound types is sensitive to the technical approach used (reviewed in Pantigoso et al., 2021; Salem et al., 2022).
*Assess relationships between diel or circadian exudation cycles and wider rhizosphere processes*: Whilst researchers have started to analyze the effect of core circadian oscillator genes on rhizomicrobiome composition (Hubbard et al., 2018, 2021; Newman et al., 2022; Staley et al., 2017), work is needed to *directly* assess rhythmic metabolic interactions between root exudates and rhizosphere microbial communities (Figure 2b,c). One approach to studying these potential rhythmic interactions could be to use microfluidics (Massalha et al., 2017).

## CIRCADIAN REGULATION OF METABOLISM ACROSS PHOTOSYNTHETIC ORGANISMS

**Figure 3:**
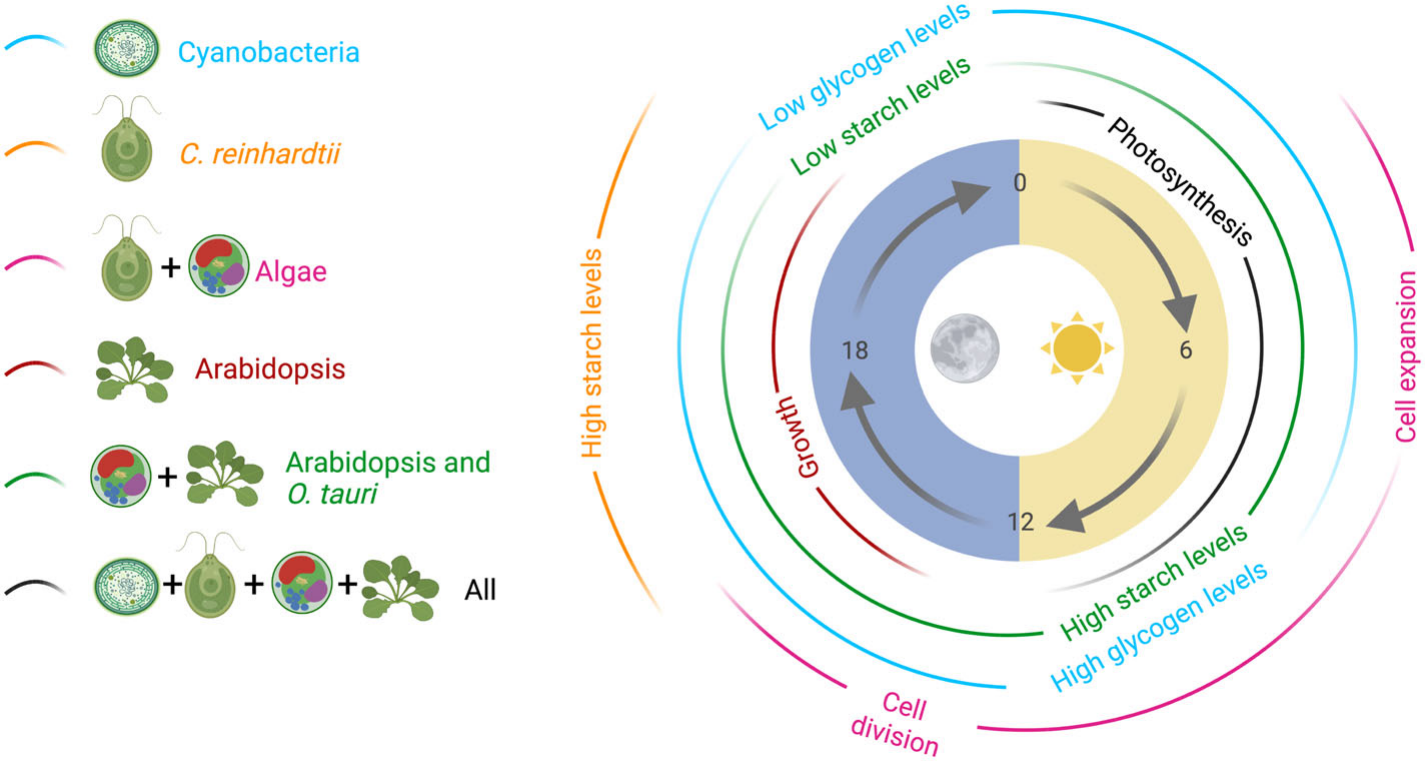
The circadian oscillator impacts metabolism across photosynthetic organisms. The diagram summarizes the times at which key metabolic processes occur in representative models for the investigation of circadian regulation in photosynthetic organisms. The diagram also indicates areas of similarity and differences between each group of organisms.

Circadian regulation provides a competitive advantage for Arabidopsis, cyanobacteria, and rodents (Dodd et al., 2005; Ouyang et al., 1998; Spoelstra et al., 2016; Woelfle et al., 2004). There are also circadian rhythms in photosynthetic eukaryotes that diverged from flowering plants at relatively early stages of plant evolution (Linde et al., 2017). In this section, we compare characteristics of the circadian regulation of metabolism in photosynthetic model organisms to explore conserved and differing aspects of the role of the circadian clock in metabolic regulation (Figure 3). We make comparisons of the circadian regulation of metabolism between Arabidopsis and cyanobacteria, as well as with the algae *Chlamydomonas reinhardtii* and *Ostreococcus tauri*. Despite its substantially different circadian clock structure, there are intimate connections between the circadian clock and metabolism in cyanobacteria, with many parallels in photosynthetic eukaryotes. Whilst *C. reinhardtii* is a freshwater biflagellate unicellular alga that lives mostly in wet soil and is motile (Sasso et al., 2018), *O. tauri* is a marine picoalga and the smallest free‐living eukaryote, moving passively with the ocean currents (Courties et al., 1994; Keeling, 2007). The contrasting environments and cell structure make these algal species good models to examine the circadian regulation of metabolism within unicellular organisms within the green lineage.

### Circadian regulation of metabolism in cyanobacteria

The biochemistry, structural biology, biophysics, and adaptive importance of the cyanobacterial circadian clock are the best understood of any circadian system (Johnson et al., 2017). The architecture of the cyanobacterial clock is unique, because transcriptional feedback is not essential for timekeeping (Tomita et al., 2005). Instead, the circadian clock in *Synechococcus elongatus* PCC 7942 is a self‐sustained post‐translational oscillator (PTO). Within this, the proteins KaiA, KaiB, and KaiC rhythmically associate and dissociate, mediating a phosphorylation and dephosphorylation cycle (Johnson et al., 2017). A core ATP hydrolysis reaction provides the key rate‐limiting reaction of this timing system (Johnson et al., 2017). The PTO itself is embedded within and regulates a TTFL that, in turn, controls the outputs of the clock and replenishes key PTO proteins (Johnson et al., 2017). In cyanobacteria, bidirectional feedback loops tune metabolism to environmental cues, and metabolic states feedback to modulate circadian clock function (Shultzaberger et al., 2015). At a conceptual level, this is remarkably similar to photosynthetic eukaryotes (Mora‐García et al., 2017).

As many cyanobacteria are obligate photoautotrophs, their cellular metabolism is strongly influenced by light/dark cycles (Cohen & Golden, 2015). In the freshwater cyanobacterium *Synechococcus* spp. RF1 and Miami BG43511, oxygen‐sensitive nitrogen fixation is circadian regulated such that it occurs out of phase with oxygenic photosynthesis (Chen et al., 1991; Grobbelaar et al., 1986; Huang et al., 1994, Mitsui et al., 1986). PCC7942, which is not a nitrogen fixer, produces oxygen‐sensitive purine biosynthesis enzymes at night (Liu, Tsinoremas, et al., 1996). In both cases, the cyanobacterial circadian clock acts to separate metabolically‐incompatible events over time.

As with other photoautotrophs, the transition to darkness prevents cyanobacteria from acquiring energy. When cyanobacteria such as PCC7942 consume energy reserves in darkness, ATP levels become depleted (Rust et al., 2011), all but essential transcription and translation are suspended, and many metabolic processes shut down (Hosokawa et al., 2011). Therefore, although darkness impacts PCC7942 metabolism considerably, circadian timing persists reliably for at least several days under constant darkness (Hosokawa et al., 2011; Tomita et al., 2005). Timekeeping in darkness is supported metabolically, in part because ATP hydrolysis by KaiC has extremely low net ATP consumption (15 ATP molecules per day, per KaiC molecule) (Terauchi et al., 2007). The overall number of ATP molecules hydrolyzed by KaiC is probably greater (Ito et al., 2009), but KaiC autodephosphorylation partly regenerates ATP used to phosphorylate KaiC (Egli et al., 2012).

Low net ATP hydrolysis likely contributes to the relative insensitivity of the cyanobacterial clock to metabolic fluctuations, a property known as metabolic compensation (Johnson & Egli, 2014; Pittendrigh & Caldarola, 1973). ATP reserves, which drive the PTO of the circadian clock, decline by up to 50% within 8 h of the onset of darkness (Rust et al., 2011). KaiC phosphorylation rhythms nonetheless continue for 2–3 days under conditions of constant darkness (Tomita et al., 2005; Xu et al., 2000). Even *in vitro* reconstituted oscillator rhythms are extremely robust to variation in the concentration of ATP (Rust et al., 2011). Modeling indicates that intertwining of the PTO and TTFL confers robustness to noise generated by temperature or metabolic fluctuations (Qin et al., 2010). As each is influenced by different types of perturbation, they compensate for one another to maintain resilient timekeeping (Qin et al., 2010). Simulations have shown that whilst PTO rhythmicity alone is robust at low growth and protein dilution rates, when cell doubling time drops below the 24 h clock period and protein dilution rate increases, the PTO ceases to function well (Zwicker et al., 2010). Under these conditions, the TTFL is required to maintain robust circadian rhythms, and conversely, the PTO dramatically enhances the robustness of the TTFL at low growth and protein dilution rates (Zwicker et al., 2010).

The circadian phase of the cyanobacterial clock is shifted by changes in ATP concentration, with a transient reduction in the ATP concentration, to simulate the effect of darkness on ATP supply, providing an entrainment cue (Rust et al., 2011). Other metabolic cues also entrain the cyanobacterial clock. Such metabolic entrainment is, in part, effected by changes in cellular redox state, through binding by KaiA of intracellular plastoquinone, which is oxidized rapidly in darkness (Wood et al., 2010) and/or the bacteriophytochrome‐like protein CikA (Schmitz et al., 2000). Thus, fluctuations in photosynthetic and catabolic activity, which impact ATP levels and cellular redox state, also participate in metabolic entrainment in PCC7942. This may occur through the direct influence of [ATP]:[ADP] on KaiC phosphorylation status (Rust et al., 2011), and blocking of the stimulation of KaiC by KaiA by oxidized quinones (Kim et al., 2012).

Robust diel rhythms of metabolism occur in cyanobacteria. Multiple cyanobacteria, including PCC7942, accumulate glycogen during the day due to CO_2_ fixation, and consume it during the night (Figure 3) (Diamond et al., 2015; Osanai et al., 2005; Schneegurt et al., 1994; Yang et al., 2002). Under constant light conditions, there is global circadian regulation of transcript abundance in PCC7942 (Vijayan et al., 2009), which has two distinct phases; subjective dusk (class 1) and subjective dawn (class 2) (Liu et al., 1995). In general, catabolic metabolic pathways are encoded within class 1 genes, whilst anabolic pathways are encoded by almost exclusively class 2 genes (Diamond et al., 2015). Under light/dark cycles, systems for oxygenic photosynthesis are activated during the day and respiratory metabolism during the night (Guerreiro et al., 2014; Stöckel et al., 2011).

The environment can drive cycles of glycogen accumulation, due to redox sensitivity of enzymes involved in glycogen metabolism (Díaz‐Troya et al., 2014). Glycogen levels also oscillate in PCC7942 under constant light conditions, and some clock mutants have arrhythmic glycogen accumulation (Figure 3) (Pattanayak et al., 2014). In another isolate of *S. elongatus*, γ‐glutamyl peptides accumulate at night and are thought to act as amino acid stores for consumption during the day (Jaiswal & Wangikar, 2020). C:N:P stoichiometry also changes over diel cycles, and *Synechococcus* might purge carbon during the night to retain cellular nutritional balance (Lopez et al., 2016). However, little work has been done to investigate the potential circadian regulation of nitrogen storage or carbon purging dynamics. Nevertheless, the PCC7942 circadian oscillator influences glycogen accumulation rhythms by modulating the balance between the Calvin cycle (anabolic metabolism) and the oxidative pentose phosphate pathway (OPPP; catabolic metabolism) under diel growth conditions (Diamond et al., 2015).

Output from the PCC7942 circadian clock is translated into gene expression patterns by the SasA‐RpaA two‐component system (Takai et al., 2006). SasA phosphorylates RpaA, a process enhanced in the presence of KaiC (Iwasaki et al., 2000). SasA is also dephosphorylated by CikA, which is stimulated in the presence of KaiBC (Iwasaki et al., 2000), producing oscillations in RpaA phosphorylation. The circadian output transcriptional regulator RpaA binds >170 gene targets, many of which function in night‐time metabolic processes such as glycogen degradation, glycolysis, and the OPPP (Markson et al., 2013). Whilst the circadian oscillator is dispensable for carbon and glycogen catabolism during the night‐time of light/dark cycles, KaiC output inhibits RpaA, preventing catabolic gene expression and blocking night‐time metabolic processes in the morning (Diamond et al., 2015). A decrease in inhibitory oscillator output during the day is then thought to allow RpaA to activate class 1 carbon catabolic genes closer to dusk (Diamond et al., 2015).

Surprisingly, a ∆*kaiC* mutant accumulates larger glycogen pools early in the light period of light/dark cycles (Diamond et al., 2015). This is counter to expectation given the inhibitory influence of KaiC on RpaA, and the fact that accumulation of transcripts involved in glycogen and carbon catabolism is greater during the subjective morning under constant light conditions (Ito et al., 2009). GlgC, which converts glucose‐1‐phosphate to the glycogen biosynthesis precursor ADP‐glucose, is allosterically activated by a reducing cellular environment and the photosynthetic product 3‐phosphoglycerate (Ballicora et al., 2003; Díaz‐Troya et al., 2014). Upon exposure to light, glycogen stores are low and photosynthesis active, thus glycogen synthesis proceeds and upregulation of catabolic genes has little influence on glycogen levels. Upon the onset of darkness, glycogen content is high and GlgC allosterically inactive. Only in darkness is transcriptional activation by RpaA a primary driving factor in glycogen catabolism (Diamond et al., 2015).

Transcriptional activation by RpaA of pyridine nucleotide transhydrogenase subunits A and B (*pntA* and *pntB*) may indirectly regulate Calvin cycle protein 12 (CP12), which is a redox‐sensitive master regulator of the Calvin cycle conserved between cyanobacteria and plants (Gontero & Maberly, 2012; Tamoi et al., 2005). PntAB allows the interconversion of NADP(H) to NAD(H), and when inhibition of RpaA by KaiC is relieved and these genes transcribed, the high NADP(H)/NAD(H) ratio present during active photosynthesis decreases and CP12 becomes activated. The clock‐mediated inhibition of primary metabolism in the morning is also thought to participate in this process, permitting secondary biosynthetic processes and plastoquinone accumulation (Diamond et al., 2015). Plastoquinone accumulation supports photosynthesis during the light period but also resets the clock (Diamond et al., 2015). Therefore, clock outputs promote the synthesis of compounds that regulate circadian timekeeping, as for Arabidopsis (Haydon et al., 2013; Webb et al., 2019).

Disruption of the SasA‐RpaA output pathway severely impairs growth under light/dark cycles (Boyd et al., 2013; Takai et al., 2006), as does disruption of key glycogen degradation and OPPP genes (Doolittle & Singer Richard, 1974; Scanlan et al., 1995). For example, a *rpaA* null mutant becomes non‐viable after several hours in darkness (Diamond et al., 2017; Puszynska & O'Shea, 2017). Reactive oxygen species (ROS) accumulate during the day and metabolic stability is lost during the night in this *rpaA* mutant (Diamond et al., 2017; Puszynska & O'Shea, 2017). RpaA‐mediated activation of genes encoding OPPP enzymes is necessary to maintain NADPH balance in the absence of photosynthesis, therefore having a critical role in night‐time metabolic stability (Diamond et al., 2017). Thus, RpaA also exerts an important control over cellular redox balance. Interestingly, ROS accumulation patterns in a *rpaA* mutant suggest that clock‐mediated and other rhythmic processes, such as those of peroxiredoxins (Edgar et al., 2012), may interact to control the cellular redox state. Redox modifications to metabolic pathways are pervasive in cyanobacteria (Ansong et al., 2014; Guo et al., 2014) and drive metabolic shifts in both cyanobacteria and plants (Díaz‐Troya et al., 2014; Lindahl & Kieselbach, 2009; Nikkanen & Rintamäki, 2014; Tamoi et al., 2005).

Further post‐translational modifications in *S. elongatus* affect metabolic activities such as photosynthesis (Yang et al., 2019), central carbon metabolism including the OPPP (Jablonsky et al., 2016), and nitrogen metabolism (Han et al., 2022). However, their relative activities over diel cycles and potential roles in circadian control of metabolism remain unknown. Intriguingly, cyclic di‐adenosine monophosphate (c‐di‐AMP) has been implicated in diel metabolic regulation in cyanobacteria (Selim et al., 2021). The carbon‐sensor protein SbtB in complex with c‐di‐AMP interacts specifically with the glycogen‐branching enzyme GlgB (Selim et al., 2021). SbtB mutants have impaired daytime glycogen synthesis, and impaired night‐time survival (Selim et al., 2021), but how this might integrate with circadian regulation remains to be identified.

### Circadian regulation of metabolism in *C. reinhardtii*

The *C. reinhardtii* circadian oscillator is structured around the *RHYTHM OF CHLOROPLAST* (*ROC*) genes (Matsuo et al., 2008). ROC15, ROC114, ROC40, ROC66, and ROC75 form the core circadian oscillator, with several of these proteins harboring domains homologous to Arabidopsis oscillator transcription factors (Matsuo et al., 2020; Matsuo & Ishiura, 2010; Ryo et al., 2016; Serrano et al., 2009). As with Arabidopsis, primary metabolism and protein synthesis are temporally aligned with the diel cycle to permit diurnal cell growth and nocturnal cell division (Strenkert et al., 2019; Zones et al., 2015). Transcripts associated with carbon metabolism are rhythmic, reflecting the cyclic patterns of photosynthetic anabolism and nocturnal catabolism (Strenkert et al., 2019). As with Arabidopsis, there are diel rhythms of starch accumulation in *C. reinhardtii*. However, peak starch levels occur in the middle of the night under conditions where acetate and CO_2_ are supplied as carbon sources (Ral et al., 2006), and this occurs with a self‐sustained circadian rhythm (Figure 3) (Klein, 1987; Thyssen et al., 2001).

An interesting feature of *C. reinhardtii* is that it can complement respiratory catabolism with fermentation, yielding NADH to generate energy. The peak expression of genes associated with starch‐fed fermentation under aerobic conditions and in the presence of CO_2_ occurs at night (Strenkert et al., 2019). Collectively, this suggests a diversified strategy to fuel growth in *C. reinhardtii* through both oxidative respiration and fermentation. The similar peak phase of starch metabolism‐ and fermentation‐related transcripts might indicate that circadian regulation acts upon these metabolic pathways to optimize carbon usage in newly divided cells. The temporally opposing growth times of *C. reinhardtii* (day) and Arabidopsis (night) might ensure there is starch available to support growth at specific times of the day. Even though both species store starch, their different times of peak starch content reflect diversity in the circadian regulation of metabolism.

### Circadian regulation of metabolism in *O. tauri*

*Ostreococcus tauri* is the smallest known photosynthetic eukaryote, with minimal cell and genomic content that is reflected in the simplified nature of its circadian oscillator (Corellou et al., 2009; Courties et al., 1994). The circadian oscillator comprises a single regulatory loop involving homologs of Arabidopsis *CCA1* and *TOC1* that harbor well‐conserved PRR, CCT, and MYB‐like domains (Corellou et al., 2009; Noordally & Millar, 2015). As with Arabidopsis, *OtTOC1* peaks around dusk, whereas *OtCCA1* peaks around the middle of the night, which is earlier than Arabidopsis *CCA1* (Corellou et al., 2009). Up to 98% of the *O. tauri* transcriptome is rhythmic under diel cycles, including transcripts encoding proteins involved in essential processes such as cell division, genome replication, and photosynthesis (de los Reyes et al., 2017; Kay et al., 2021; Monnier et al., 2010). Transcripts associated with starch metabolism are extensively circadian regulated in *O. tauri*; this includes transcripts encoding chloroplast phosphoglucoisomerase (PGI), PGM, starch synthase GBSSI, the small subunit of AGPase, GWD, and SEX4, which are rhythmic under diel cycles (Sorokina et al., 2011). However, the relationship between circadian regulation of transcript and protein abundance in *O. tauri* is only partial, suggesting that these changes in transcript levels might not produce rhythms in protein abundance or metabolic pathway activity (Kay et al., 2021), in a similar manner to Arabidopsis (Figure 1c). In *O. tauri*, there is circadian regulation of a small number of chloroplast‐encoded proteins associated with photosynthesis, transcription and translation (Kay et al., 2021), supporting the notion that circadian regulation of photosynthetic metabolism and chloroplast gene expression is conserved across the green lineage. This includes a circadian rhythm in the protein abundance of a nuclear‐encoded and chloroplast‐localized sigma factor (SIG6), which is reminiscent of the circadian regulation of sigma factors in Arabidopsis that can influence photosynthetic metabolism (Cano‐Ramirez et al., 2023; Kay et al., 2021; Noordally et al., 2013). This study identified only two mitochondrial proteins with circadian rhythms in abundance (Kay et al., 2021). Although this could suggest less extensive circadian regulation of mitochondrial processes, it does not exclude the possibility of extensive circadian regulation at the post‐translational level.

Transcripts associated with starch metabolism are extensively circadian regulated in *O. tauri*. This includes transcripts encoding chloroplast PGI, PGM, starch synthase GBSSI, the small subunit of ADP‐Glc pyrophosphorylase (AGPase), GWD, and SEX4, which are rhythmic under diel cycles (Sorokina et al., 2011). The abundance of transcripts encoding the small subunit of AGPase is also rhythmic under light/dark cycles in *C. reinhardtii*, and under free‐running conditions in Arabidopsis (Covington et al., 2008; Harmer et al., 2000; Ral et al., 2006). In *C. reinhardtii*, the high nocturnal starch content corresponds with peak transcript abundance and protein activity of the small subunit of AGPase (Ral et al., 2006). This suggests conservation between *O. tauri*, *C. reinhardtii* and Arabidopsis in the circadian regulation of an enzyme that catalyzes a rate‐controlling step in starch anabolism (Ballicora et al., 2003; Okumura et al., 2016; Smith et al., 2004; Smith & Stitt, 2007; Smith & Zeeman, 2020), despite the differences in circadian clock architecture. Key rate‐limiting enzymes associated with primary metabolism appear to be conserved points of circadian regulation within the green lineae, and perhaps were selected for early in the evolution of photosynthetic eukaryotes.

## COMPARISON BETWEEN CIRCADIAN REGULATION OF METABOLISM IN PLANTS AND NON‐PHOTOSYNTHETIC ORGANISMS

Here, we extend our comparison of the circadian regulation of metabolism in plants to non‐photosynthetic organisms, including a prokaryote (*Bacillus subtilis*), a filamentous fungus (*Neurospora crassa*), and mammals. Our goal is to identify shared and differing aspects of the circadian regulation of metabolism between plants and models representing non‐photosynthetic branches of the tree of life.

### Comparison with circadian regulation of metabolism in non‐photosynthetic bacteria

Circadian rhythms have been reported in the non‐photosynthetic bacteria *Klebsiella aerogenes* (Paulose et al., 2016) and *B. subtilis* (Eelderink‐Chen et al., 2021). Little is known about the molecular architecture and physiological functions of the circadian clocks in these species, but their circadian programs might integrate closely with metabolic processes. In *B. subtilis*, circadian rhythms are detectable under specific nutrient conditions, suggesting that the metabolic environment is important for the regulation of the *B. subtilis* circadian clock (Eelderink‐Chen et al., 2021). Similarly, the circadian program of *K. aerogenes* is influenced by the presence of exogenous melatonin (Paulose et al., 2016). In contrast to photosynthetic organisms, nutrient availability for non‐photosynthetic bacteria can depend on metabolic activity of environmental or host organisms, such as plants, which could themselves be circadian regulated (Sartor et al., 2019). For example, *B. subtilis* forms biofilms on plant roots (Arnaouteli et al., 2021; Blake et al., 2020), responds to root exudates (Allard‐Massicotte et al., 2016; Chen, Cao, et al., 2012), and there might be circadian regulation of root exudation (Lu et al., 2021; Newman et al., 2022), suggesting that metabolic processes could mediate interactions between circadian programs in micro‐ and macro‐organisms (Figure 2c) (Sartor et al., 2019).

### Comparison with circadian regulation of metabolism in *N. crassa*

*Neurospora crassa* is the best‐understood fungal circadian clock model, with circadian regulation of up to 40% of the transcriptome (Hurley et al., 2014; Sancar et al., 2015). The core circadian oscillator is formed from the *frq* gene together with the GATA transcription factors White Collar 1 (WC‐1, a blue light photoreceptor) and White Collar 2 (WC‐2) which, through their PAS domains, form a heterodimeric White Collar Complex (WCC) (Baker et al., 2012; Cha et al., 2015; Dunlap & Loros, 2017; Liu & Bell‐Pedersen, 2006; Tseng et al., 2012). *Neurospora* and Arabidopsis share principles of circadian oscillator regulation such as control of subcellular localization, phosphorylation, and protein turnover (Baker et al., 2012; Harmer et al., 2001; Young & Kay, 2001). Circadian regulation in *Neurospora* participates in responses to long‐term nutritional stress, reorganizing the transcriptome in response to nutrient availability with clock mutants having reduced growth once nutrients become available (Szőke et al., 2023). Similarly, there is reduced growth potential in Arabidopsis circadian clock mutants that appears linked to temporal management of carbohydrate resources (Dodd et al., 2005; Graf et al., 2010).

Metabolic compensation forms an important part of metabolic regulation in *Neurospora*. Net circadian period stabilization occurs under varying metabolite concentrations, which is controlled mainly by auxiliary clock elements CSP1, RCO1, an ATP‐dependent RNA helicase, PERIOD 1 (PDR1) acting with pathways influencing mRNA stability, chromatin modification, and polyadenylation (Emerson et al., 2015; Gyongyosi et al., 2017; Kelliher et al., 2023; Olivares‐Yanez et al., 2016; Sancar et al., 2012). Although this is not dissimilar from metabolic alterations in circadian oscillator dynamics in Arabidopsis (Haydon et al., 2013; Webb et al., 2019), a key difference is that the Arabidopsis oscillator is entrained by the products of photosynthesis which depends on the external factor of light.

Amongst many other transcription factors, the WCC modulates expression of the glucose‐dependent gene *conidial separation 1* (*csp1*), whose product is a circadian transcriptional repressor mainly targeting metabolism‐associated genes (Sancar et al., 2011, 2015). The WCC, through its accessory clock components CSP1 and VOS1, exhibits transcriptional control of *glycogen synthase* and *glycogen phosphorylase* leading to rhythmic accumulation and degradation of glycogen, respectively (Baek et al., 2019). This is conserved conceptually with Arabidopsis, where the circadian oscillator contributes to starch turnover dynamics (Graf et al., 2010; Seki et al., 2017).

### Comparison with circadian regulation of metabolism in mammals

Mammals have cell‐ and organ‐specific circadian oscillators that are synchronized locally, and hierarchically controlled by the suprachiasmatic nucleus (SCN) (Chaix et al., 2016; Welsh et al., 2010). This is similar to the notion that Arabidopsis shoot apex or leaf circadian oscillators have a role in regulating circadian oscillators in other tissues (James et al., 2008; Takahashi et al., 2015). However, although the SCN is the synchronizing center of circadian rhythms for the entire organism, a specific peripheral organ (the liver) has a key role in glycogen carbon storage and glucose release (Mohawk et al., 2012; Panda, 2016; Welsh et al., 2010). In plants, energy intake occurs strictly during the daytime, when there is light available for photosynthesis and sucrose synthesis, and fasting occurs during the night when starch reserves are mobilized. In mice fed *ad libitum*, most food is consumed during the night, but this has flexibility depending on the calorific content of the food (Panda, 2016). Thus, circadian metabolic regulation in both Arabidopsis and mammals incorporates feeding‐fasting cycles, although their underlying biochemistry is different. In Arabidopsis, there are multiple points of circadian regulation of daytime sucrose anabolism (Covington et al., 2008; Harmer et al., 2000; Krahmer et al., 2022; Scandola et al., 2022), as well as the nocturnal starch catabolism yielding sucrose (Graf et al., 2010; Smith & Zeeman, 2020), with this sucrose entraining the circadian oscillator (Haydon et al., 2013). In mammals, feeding and nutrients act as zeitgebers, and their timing can be temporally aligned with or be mismatched from the light/dark cycle depending on the species and food composition (Asher & Sassone‐Corsi, 2015; Poggiogalle et al., 2018).

Another common point of circadian metabolic regulation in Arabidopsis and mammals is the pervasiveness of the circadian control of key enzymes involved in primary metabolism, such as the rate‐limiting AGPase associated with starch synthesis in Arabidopsis and the glycogen synthase kinase (GSK3) in mammals (Bläsing et al., 2005; Covington et al., 2008; Harmer et al., 2000; Panda, 2016; Usadel et al., 2008; Yin et al., 2006). Circadian regulation also acts upon signaling molecules that coordinate metabolism in both Arabidopsis and mammals. In mammals, insulin production and sensitivity are circadian regulated (Marcheva et al., 2013; Perelis et al., 2015; Poggiogalle et al., 2018; Van Cauter et al., 1997), and glucagon, associated with glucose release, is circadian regulated through protein kinase A (Narasimamurthy et al., 2012; Panda, 2016; Zhang et al., 2010). The analogous metabolic signal to insulin in plants is Tre6P, which acts as a negative regulator of sucrose concentrations in Arabidopsis (Figueroa & Lunn, 2016). Transcripts encoding TREHALOSE‐6‐PHOSPHATE SYNTHASE1 (TPS1) and Tre6P phosphatase (TPP) are rhythmic (Covington et al., 2008) and the concentration of Tre6P has diel oscillations that anticipate dawn and dusk (Annunziata et al., 2018; Wahl et al., 2013). Therefore, the concentration of Tre6P might be regulated directly by the circadian oscillator, or indirectly by the levels of sucrose and/or starch degradation.

## CONCLUSIONS AND FUTURE PROSPECTS

The considerable impact of the circadian regulation of metabolism on plant performance makes understanding this regulatory system crucial to the exploitation of knowledge of circadian regulation to optimize crop performance (Steed et al., 2021). One constraint upon the extension of findings from Arabidopsis to crops is the diversity of metabolic processes across the green lineage, including differences in molecules used for nocturnal energy storage. However, we argue that the over‐arching principles of the circadian control of metabolism are well conserved, so it should be possible to translate knowledge about circadian regulation of metabolism from model species to important crops. Since the products of metabolism impact the interactions between plants and all domains of life within an ecosystem, the circadian regulation of metabolism might contribute to temporal alignment between plants and other interacting organisms within ecosystems (Box 2).

Box 2 **Open questions**

- What are the relative contributions of transcriptional control and post‐translational regulation to the circadian regulation of metabolism?
- How does the circadian regulation of root exudation shape the rhizomicrobiome?
- What mechanisms underlie the circadian regulation of photosynthetic light harvesting, and what is the role of circadian regulation in C_4_ photosynthesis?
- What is the role of metabolites in achieving temporal alignment between plants and interacting organisms?

## CONFLICT OF INTEREST

The authors declare no conflicts of interest.
